# Diagnostics Increases Visibility

**DOI:** 10.3390/diagnostics14141554

**Published:** 2024-07-18

**Authors:** Andreas Kjaer

**Affiliations:** 1 Department of Clinical Physiology and Nuclear Medicine, Copenhagen University Hospital—Rigshospitalet, 2100 Copenhagen, Denmark; akjaer@sund.ku.dk; 2 Cluster for Molecular Imaging, Copenhagen University Hospital—Rigshospitalet, Department of Biomedical Sciences, University of Copenhagen, 2200 Copenhagen, Denmark

It is with great pleasure that we announce that our journal has recently received the 2023 CiteScore of 4.7. Diagnostics was indexed by Scopus in 2019 and received its first CiteScore of 1.1 in 2019. Since then, we have seen a steady increase with CiteScores of 1.4, 2.4, and 3.6 in 2020, 2021, and 2022, respectively. This puts Diagnostics in Q2 in the categories “Clinical Biochemistry” and “Internal Medicine”.

In terms of impact factor (Clarivate), we see a stable level of 3–4 since 2019, with 3.0 in 2023. This is a slight decrease from 2022, but this was observed for the majority of journals within our subject category “Medicine, General and Internal” probably due to an extraordinary level observed in relation to the COVID-19 pandemic. Accordingly, we increased our ranking within our category from Q2 to Q1 and are now ranked 58th out of 325 journals indexed in the “Medicine, General and Internal” category.

Taken together, Diagnostics has established itself as a highly visible and highly cited journal for the scientific community. This has happened as we have focused on developing and elevating the importance of Diagnostics to new levels. Accordingly, we have seen an ever-increasing number of submissions in all of our paper categories, and in 2023 the total number of submissions was 8173. The overall acceptance rate is currently 46%.

The positive development of Diagnostics relies heavily on the continued support of researchers and the scientific community, as well as the excellent work performed on a daily basis by the Editorial Board, the Editorial Office staff, and the Editor.

I would like to thank all those who have contributed and will continue to contribute to the ongoing success of Diagnostics.

